# Influence of the heme distal pocket on nitrite binding orientation and reactivity in Sperm Whale myoglobin

**DOI:** 10.1042/BCJ20200596

**Published:** 2021-02-26

**Authors:** Wilford Tse, Nathan Whitmore, Myles R. Cheesman, Nicholas J. Watmough

**Affiliations:** 1 Department of Biological Sciences, Centre for Molecular and Structural Biochemistry, University of East Anglia, Norwich Research Park, Norwich NR4 7TJ, U.K.; 2 Department of Chemistry, University of East Anglia, Norwich Research Park, Norwich NR4 7TJ, U.K.

**Keywords:** heme, magnetic circular dichroism spectroscopy, myoglobin, nitric oxide, nitrite

## Abstract

Nitrite binding to recombinant wild-type Sperm Whale myoglobin (SWMb) was studied using a combination of spectroscopic methods including room-temperature magnetic circular dichroism. These revealed that the reactive species is free nitrous acid and the product of the reaction contains a nitrite ion bound to the ferric heme iron in the nitrito- (O-bound) orientation. This exists in a thermal equilibrium with a low-spin ground state and a high-spin excited state and is spectroscopically distinct from the purely low-spin nitro- (N-bound) species observed in the H64V SWMb variant. Substitution of the proximal heme ligand, histidine-93, with lysine yields a novel form of myoglobin (H93K) with enhanced reactivity towards nitrite. The nitrito-mode of binding to the ferric heme iron is retained in the H93K variant again as a thermal equilibrium of spin-states. This proximal substitution influences the heme distal pocket causing the p*K*_a_ of the alkaline transition to be lowered relative to wild-type SWMb. This change in the environment of the distal pocket coupled with nitrito-binding is the most likely explanation for the 8-fold increase in the rate of nitrite reduction by H93K relative to WT SWMb.

## Introduction

Heme containing nitrite reductases play a significant role in the transformation of nitrite to both nitric oxide and ammonia in animals, plants and bacteria. One of the most extensively characterised is the pentaheme NrfA from *Escherichia coli* that catalyses the six-electron reduction in nitrite to ammonium with no evidence for release of the putative intermediate NO [1–4]. On the contrary, the enzyme can actually catalyse the reduction in NO to ammonium, albeit at significantly lower rates [4].

A distinguishing feature of the *E.coli* NrfA [1], as well as the structurally well characterised enzymes from the ε-proteobacteria *Sulfurospirillum deleyianum* [5] and *Wolinella succinogenes* [6] and δ-proteobacteria *Desulfovibrio desulfuricans* [7] and *D. vulgaris* [8], is a highly unusual proximal lysine ligand to the active site *c*-type heme. This raises questions as to why this ligation is preferred to the far more common histidine. The lysine may have a fundamental role in the activation of nitrite towards reduction or it may impact on one of the NrfA specific characteristics such as the ability to reduce NO in addition to nitrite. However, this remains unclear in the absence of a well-characterised nitrite reductase with histidine coordinated heme for comparison.

Nitrite is an ambidentate ligand that can bind to metal centres either via its nitrogen atom (nitro-orientation) or via one of its oxygen atoms (nitrito-orientation). Low temperature X-ray crystallographic studies of the interaction between nitrite and NrfA from *W. succinogenes* reveals binding to the ferric active site heme in the nitro-orientation [9]. This orientation is maintained by hydrogen bonds formed between the oxygen atoms of nitrite and two conserved distal pocket residues; Arg-106 and His-256 (*E. coli* numbering). Substitution of the conserved histidine with asparagine in the enzymes from *W. succinogenes* and *E. coli* abolished nitrite reduction [3]. Interestingly the H256A variant of *E. coli* NrfA has a greater affinity for nitrite than the wild-type enzyme, but in the absence of reliable spectroscopic signatures of nitro- and nitrito-bound ferric heme it was not possible to resolve the impact of the substitution on binding orientation.

An opportunity to probe the influence of heme pocket architecture on the binding and activation of nitrite is provided by the vertebrate myoglobins, the primary function of which is short-term oxygen (O_2_) storage to sustain respiration [10]. However, these proteins can moonlight and reduced myogobins can also transform nitrite (NO_2_^−^) to nitric oxide (NO) [11]:

$$\text{N}{\text{O}}_{2}^{-}+{\text{e}}^{-}+2{\text{H}}^{+}\to \text{NO}+{\text{H}}_{2}\text{O}$$

This secondary role appears to be more significant under hypoxic conditions [12] when tissues cannot use NO synthetase to generate NO, a potent vasodilator, from arginine and O_2_. Proximal histidine ligation of the heme may have evolved to serve myoglobin's primary role as an oxygen store and whilst nitrite reduction is not precluded it may be compromised. Substitution of the proximal histidine (His-93) with lysine in myoglobin would allow a direct comparison of the influence of these two ligands on nitrite binding within an active site framework that is already known to be capable of nitrite reduction.

**Figure 1.**
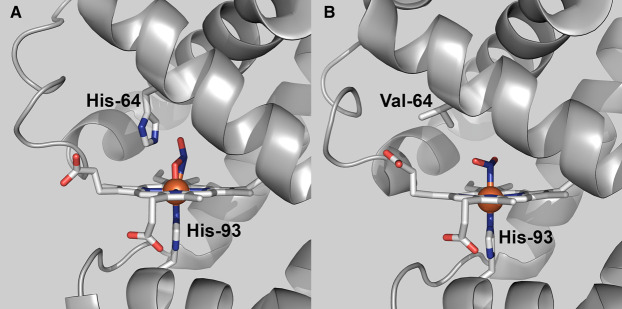
Modes of nitrite binding to ferric heme in horse heart myoglobin. (**A)** Nitrite bound to the ferric heme iron of wild-type HHMb in the nitrito (O-bound) orientation (PDB 2FRF) [13]. **(B)** Nitrite bound to ferric heme iron of the H64V variant of HHMb in the nitro (N-bound) orientation (PDB 3HEP) [16]. Images were prepared in PyMol (The PyMol Molecular Graphics System, Version 2.4.1, Schrödinger, LLC).

X-ray crystallography shows nitrite binding to the ferric heme in horse heart myoglobin (HHMb) in the nitrito-orientation and suggests that the conserved histidine residue (His-64) in the heme distal pocket plays a role in orientating the substrate to bind to the heme iron via one of its oxygen atoms (Figure 1A) [13,14]. The same mode of binding was reported for the X-ray structure of the nitrite bound form of ferric haemoglobin [15]. However, the substitution of H64 with valine in HHMb leads to nitrite adopting the alternative nitro-conformation in the crystal (Figure 1B) [16].

These observations pose two questions regarding the reactivity of vertebrate globins towards nitrite. Firstly, what is the active form of the nitrite ion in solution and is the product of its reaction with myoglobin the same nitrito-bound form observed in crystallographic experiments? Secondly does substitution of the proximal heme ligand (His-93) of myoglobin with lysine have any consequences for the reactivity of the protein towards nitrite or the mode of ligand binding? Here we describe the expression and characterisation of a novel form (H93K) of Sperm Whale myoglobin (SWMb) and show for the first time that room-temperature magnetic circular dichroism (RTMCD) spectroscopy reliably discriminates between the nitrito- and nitro-modes of binding to heme iron. This allowed us to demonstrate that both wild-type and the H93K variant of Sperm Whale myoglobin (SWMb) react with free nitrous acid (HNO_2_) to bind substrate in the nitrito-orientation. The enhanced reactivity towards nitrite exhibited by the H93K variant may be accounted for by a long-range effect on the distal pocket which manifests itself through a change in the p*K*_a_ of the water bound to the distal face of the ferric heme iron.

## Experimental

### Protein expression and purification

The *Escherichia coli* strain TB1 was transformed with a plasmid containing a synthetic gene that encodes either WT SWMb [17] or a variant in which the proximal histidine (His93) is substituted by lysine or the distal histidine (H64) with valine. Conditions for batch cell culture and subsequent purification of WT SWMb and both variants were essentially as previously described [18] with the addition of a final Superdex S-75 gel filtration step Samples used in subsequent experiments exhibited a single band on SDS–PAGE and an absorption ratio (Soret maximum/280 nm) > 3. The presence of each substitution was confirmed by mass spectroscopy.

### Spectroscopic measurements

Electronic absorption spectra were recorded on either Cary 4000 UV-visible spectrophotometer or a Hitachi U4001 spectrophotometer. The concentration of SW Mb was calculated using the following molar extinction coefficients: wild-type, ε_410_ = 1.57 × 10^5^ M^−1^ cm^−1^; H64V, ε_395_ = 1 × 10^5^ M^−1^ cm^−1^; [18].

Magnetic Circular Dichroism spectra in the visible region were recorded using a J-810 spectropolarimeter with the sample located in an 8-T magnetic field within the ambient temperature bore of an Oxford Instruments Special 1000 superconducting solenoid [19].

Continuous wave EPR spectra were recorded on either a Bruker ELEXSYS 500 spectrometer with an ER049X SuperX microwave bridge and a super high Q cavity or on an X-band ER200-D spectrometer interfaced to an ESP1600 computer. Low temperature experiments were performed using an Oxford Instruments ESR-900 helium cryostat and an ITC3 temperature controller.

Integrating the simulated EPR spectra allows the relative amounts of the high- and low-spin forms of ferric heme in myoglobin treated with nitrite at 10 K to be estimated. These estimates assume that all of the ferric heme species are represented in the CW X-band EPR spectrum recorded at 10 K. This assumption requires that all high-spin population remains in the lowest doublet, m_s_ = ±1/2, with negligible population in the m_s_ = ±3/2 and m_s_ = ±5/2 doublets that are EPR undetectable in the axial limit. The m_s_ = ±3/2 doublet lies 2D above the m_s_ = ±1/2 doublet, where D is the axial zero-field splitting parameter. D for hemes is typically 5–10 cm^−1^. At D ≈ 5 cm^−1^ the population of the m_s_ = ±3/2 doublet will be <0.5%. i.e. it is reasonable to assume that the integrated intensity of the g ≈ 6 features is indicative of the high-spin content.

### Nitrite reduction assays

The rate of nitrite reduction was measured at 25°C using a modification of the method described by Tejero and colleagues [20]. Each reaction mixture (3.0 ml) was prepared in a 3.5 ml quartz cuvette in an anaerobic glove box and contained 100 mM potassium phosphate, 30 mM sodium dithionite and 5–10 µM myoglobin, pH 7.4. Reactions were initiated by addition of sodium nitrite from an anaerobic stock solution to yield the desired final concentration of nitrite (0.05 to 1 mM). The progress of the reaction was monitored by recording changes in the Soret region (380–480 nm) of the electronic absorption spectrum.

### Reaction of nitrite with Met-myoglobin

The reaction of the various metmyoglobins with nitrite under pseudo first order conditions was measured in an Applied Photophysics Bio-Sequential DX.17MV stopped-flow spectrophotometer using a 1 cm pathlength cell. Detection at a single wavelength was with a side window photomultiplier. In this configuration, a minimum of 1000 data points were collected per experiment. Experimental traces recorded at 407 nm were analysed as single exponential decay to derive pseudo-first order rate constant (*k*_obs_) and amplitude (change in absorbance; ΔA) for the reaction.

### Data export and re-plotting

Data files from spectrometers were exported as text or csv files and re-plotted for analysis using either Origin v6.0 or QtiPlot.

## Results

### Reaction of ferric wild-type Sperm Whale myoglobin with nitrite

**Figure 2.**
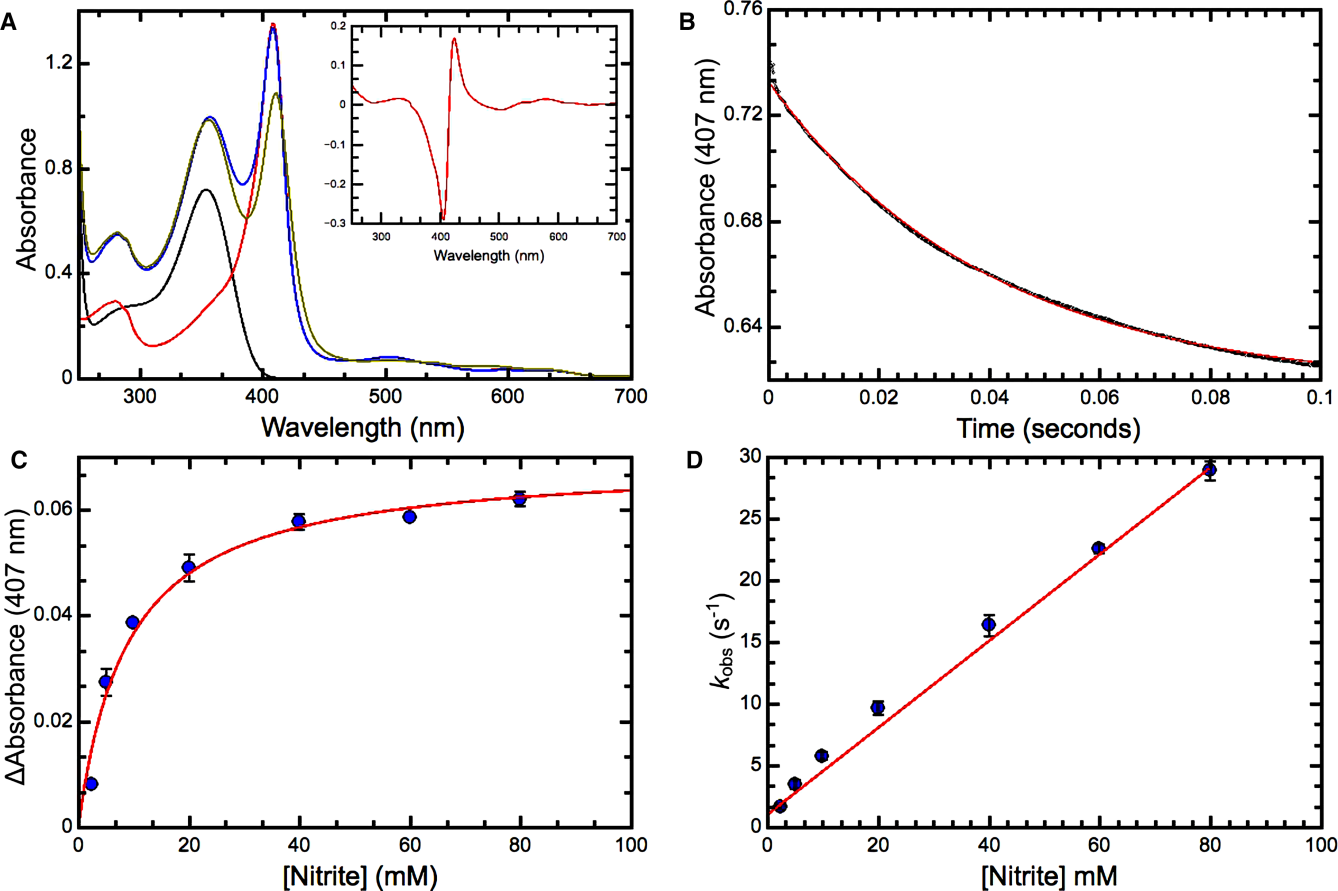
Reaction of ferric wild-type Sperm Whale myoglobin with nitrite. (**A**) UV/visible spectra of 8.8 µM ferric WT SWMb (red trace); 30 mM nitrite (black trace), 8.8 µM ferric WT SWMb + 30 mM nitrite pre-mixing (blue trace); 8.8 µM ferric WT SWMb + 30 mM nitrite post-mixing (olive trace). Inset the ferric-nitrite *minus* ferric difference spectrum. This was derived by subtracting the spectrum of 8.8 µM ferric WT SWMb + 30 mM nitrite pre-mixing (blue trace) from the spectrum of 8.8 µM ferric WT SWMb + 30 mM nitrite post-mixing (olive trace). (**B**) Progress of the reaction between 3.5 µM ferric WT SWMb and 40 mM nitrite monitored at 407 nm. Small solid black circles represent data points and the red trace is a fit to a single exponential (*k*_obs_ = 17 s^−1^). (**C**) Dependence of the amplitude ΔA of the reaction as a function of nitrite concentration. Solid blue circles are the mean of at least three independent observations and the red trace a fit to a simple binding isotherm (*K*_d_ = 8.93 mM). (**D**) Dependence of *k*_obs_ as a function of nitrite concentration. Solid blue circles are the mean of at least three independent observations and the red trace a linear fit. All reactions were carried out in 20 mM phosphate-citrate buffer pH 7.0.

The effect of nitrite binding to Ferric heme in wild-type (WT) SWMb is to cause the Soret maximum to decrease in intensity and shift from 410 nm to 416 nm (Figure 2A). Under our experimental conditions we have found no evidence of new intensity at 450 nm that is associated with nitration of the porphyrin macrocycle [21]. In the visible region, an associated decrease in absorbance at 504 nm is observed along with the emergence of a new feature at 571 nm. Nitrite binding also induces a small shift in the position of the weak feature at 633 nm to 627 nm.

The dissociation constant (*K*_d_) for nitrite binding to ferric WT SWMb was determined by rapidly mixing protein with nitrite under pseudo-first order conditions in a stopped-flow spectrophotometer [22]. The progress of the reaction was monitored via the decrease in absorbance at 407 nm that represents the biggest absorbance change in the ferric-NO_2_^−^ *minus* ferric difference spectrum (Figure 2A inset). The reaction progress curve can be fitted to a single exponential to determine the observed rate constant (*k*_obs_) and the amplitude (change in absorbance at 407 nm) for the reaction (Figure 2B) at a specified [NO_2_^−^]. The observed amplitude ΔA_obs_ at any given [nitrite] relative to the maximum change of absorbance (ΔA_max_) associated with 100% occupancy gives an indication of the fractional saturation of the heme iron by nitrite. Hence a plot of the ΔA_obs_ as a function of [nitrite] can be fitted to a simple binding isotherm (Figure 2C) to estimate the dissociation constant (*K*_d_) for nitrite binding (Table 1). Since reactions were carried out under conditions in which [nitrite] >> [SWMb], the bimolecular rate constant (*k*_+1_) for nitrite binding and the first order rate constant (*k*_−1_) that describes dissociation of the myoglobin:nitrite complex (Table 1) can be obtained from plots of the observed rate constant (*k*_obs_) vs [NO_2_^−^] (Figure 2D). When the measurements are repeated at pH 6.0 the value of *k*_+1_ increases ∼10-fold (Table 1), an observation that would be consistent with the active species being the protonated form of the nitrite ion free nitrous acid (HNO_2_).

**Table 1:** Summary of the properties of the SWMb variants used in this study

	Nitrite Binding (ferric heme)					Nitrite Reduction (ferrous heme)	
	pH	*K*_d_	*k*_+1_	*k-*_1_	*K*_eq_	*k*_nitrite_	pK^1^
Wild-type	7.0	8.93 mM	0.35 mM^−1^ s^−1^	1 s^−1^	2.68 mM	17.8 M^−1^ s^−1^	8.9
	6.0	4.66 mM	4.86 mM^−1^ s^−1^	2.56 s^−1^	0.5 mM		
H93K		1.92 mM	0.33 mM^−1^ s^−1^	0.61 s^−1^	1.84 mM	121 M^−1^ s^−1^^1^	7.8
H64V		>150 mM	nd	nd	nd	1.5 M^−1^ s^−1^^2^	

1 Based on a rate constant determined in the presence of 50 µM nitrite;

2 Based on a rate constant determined in the presence of 0.5 mM nitrite.

### Spectroscopic characterisation of the product of the reaction between ferric WT SWMb and nitrite

To characterise the reaction product, the RT-MCD spectrum of WT SWMb was recorded before and after the addition of nitrite. Although MCD probes the same electronic transitions as electronic absorption spectroscopy, it offers significant advantages. Signed bands and substantial variations in intensity of the porphyrin π–π* transitions in the UV-visible region identify, the spin- and oxidation- states of the heme iron. Furthermore, for high- and low-spin ferric states, MCD can locate charge-transfer (CT) transitions in the range 600–2000 nm that are diagnostic of the axial ligands. A single positive band (CT_LS_) appears for low-spin heme whereas the high-spin state gives rise to two bisignate features (CT_1_ and CT_2_).

**Figure 3.**
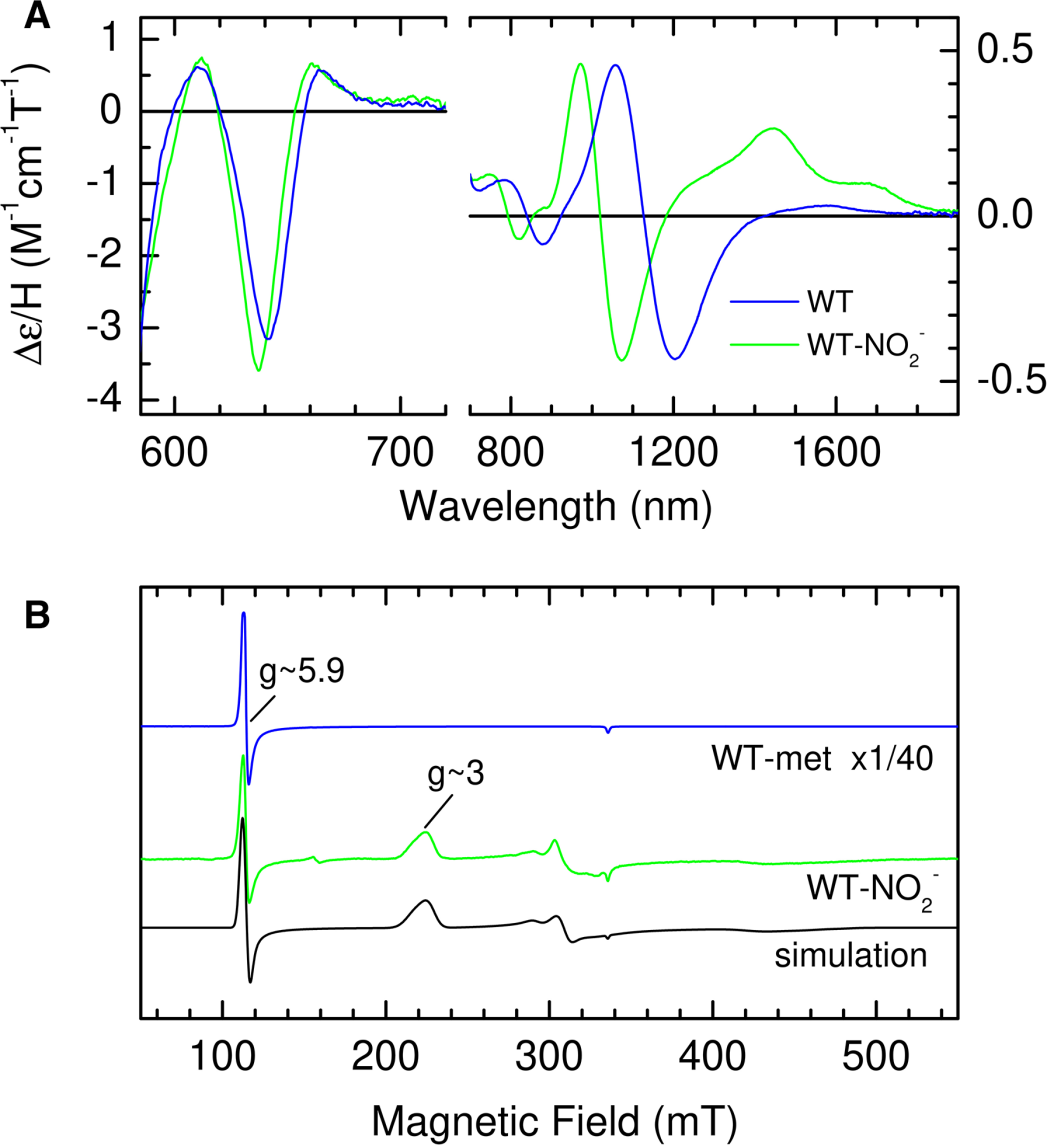
Spectroscopic analysis of the product of the reaction between ferric WT SWMb and nitrite. (**A**) nIR RT-MCD spectrum of ferric WT SWMb (790 µM) before (blue trace) and after (green trace) the addition of 100 mM nitrite. (**B**) X-band EPR spectrum of ferric WT SWMb (320 µM) before (blue trace) and after the addition of 100 mM nitrite. The spectrum of the reaction product was simulated (black trace) with three components as described in Materials and Methods.

After addition of 100 mM nitrite to WT SWMb all bands in the UV-visible region MCD spectrum remain consistent with a Ferric heme species (Supplementary Figure S1A); none of the heme becomes reduced, but the bisignate Soret feature in the MCD spectrum shifts from 409 nm to 415 nm and increases in (peak-to-trough) intensity to ≈40 M^−1^ cm^−1^ T^−1^ indicating a sub-stoichiometric high-spin to low-spin conversion (Supplementary Figure S1B). This is clearly demonstrated by the emergence in the nIR-MCD spectrum of a low-spin CT band at 1444 nm which is interpreted as arising from low-spin ferric heme with His/NO_2_^−^ axial coordination (Figure 3A). Given the saturating [nitrite] this observation cannot simply be accounted for by incomplete binding of NO_2_^−^ to form a sub-stoichiometric amount of a purely low-spin form.

There are also changes in the position of both high-spin CT bands; CT_1_ moves from 1100 nm to 1020 nm, whilst the minimum (trough) of CT_2_ moves from 642 nm to 637 nm (Figure 3A). It is important to note that there is no increase in bandwidth associated with the shift in position of CT_2_ and there is no residual trace of the 642 nm feature. Thus, the binding studies indicate complete coordination by NO_2_^−^ yet the MCD, while confirming that no unbound heme remains, shows a mixture of spin states. There is a precedent for this behaviour: when hydroxide is bound distal to histidine in heme proteins, an axial ligand field of intermediate strength places the system close to the spin-crossover and it exists in a thermal equilibrium of spin-states [23,24]. Although the ground state cannot be ascertained directly from RT-MCD, the relative amounts of each state suggest a high-spin excited state which is readily accessible at room temperature and a low-spin ground state.

The nature of the ground state was investigated using low temperature (10 K) CW-X-band EPR (Figure 3B). WT-SWMb gives rise to a typical high-spin S = 5/2 spectrum with g_xyz_ = 5.9, 5.9, 2.0 [25]. Following nitrite binding, a negligible level of high-spin features remains, and the spectrum is dominated by broad features at g ≈ 3.0 and in the region of 2.10–2.30. These are recognisable as the g_z_ and g_y_ components of low-spin ferric heme implying that, as is the case for His/HO^−^ ligated spin-mixtures [23,24], the ground state is low-spin S = 1/2 and that any high-spin form is largely depopulated at 10 K. We therefore conclude that NO_2_^−^ is bound to the iron via oxygen and by analogy with hydroxide, also an anionic oxygen ligand, exists in a similar thermal spin mixture. Close inspection of the signal at g ≈ 5.9 reveals differences in line shape compared with the unbound form. This observation is consistent with this being a trace of the high-spin component of NO_2_^−^ bound heme rather than remnants of unbound starting material. Simulation of the spectrum (Figure 3B), followed by double integration, indicates that this high-spin species represents only 2% of the total heme, confirming a low-spin ground state.

### RT-MCD spectroscopy can discriminate between the nitrito- and nitro- orientations of nitrite binding to the ferric heme

**Figure 4.**
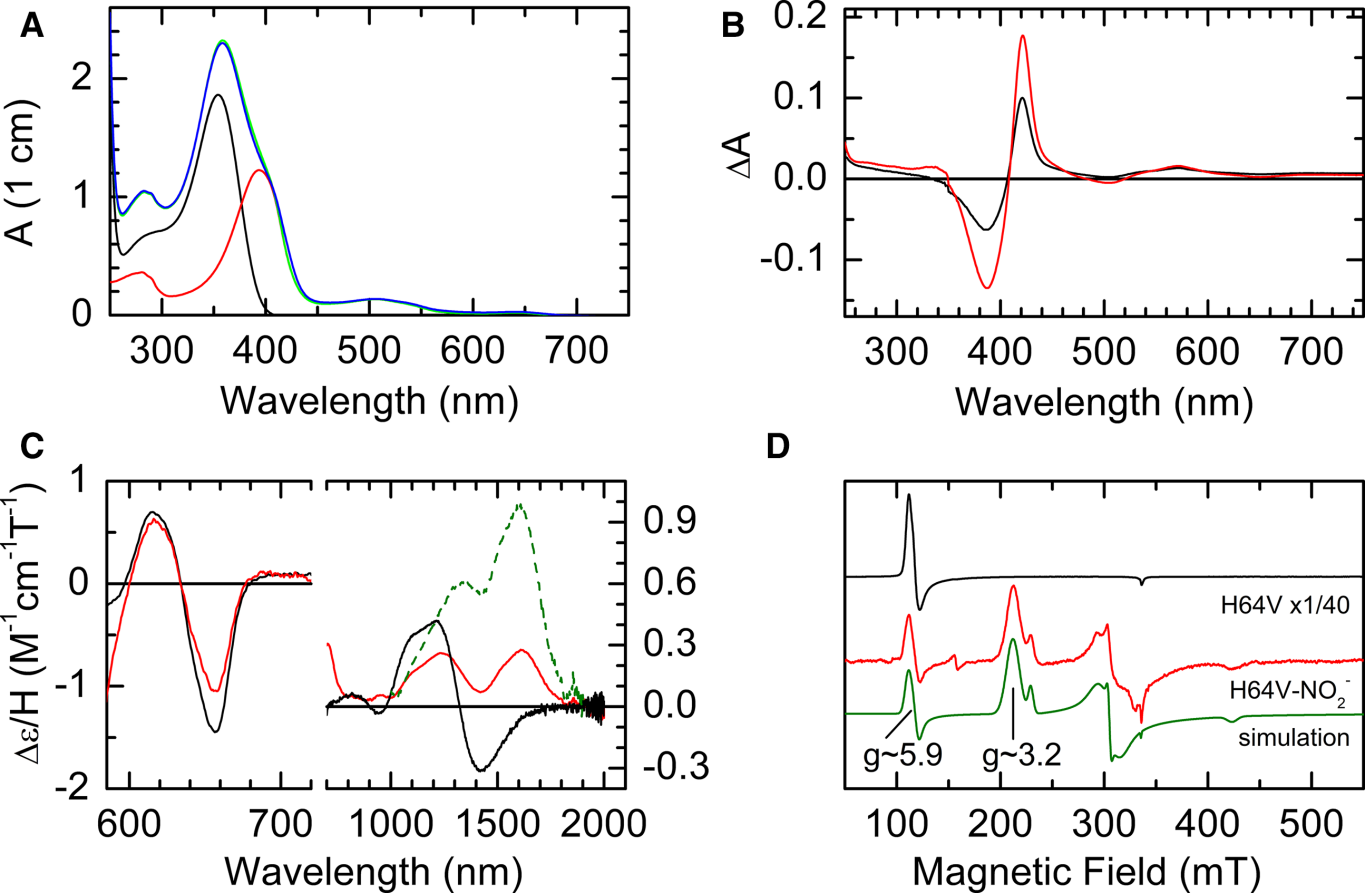
Spectroscopic analysis of the product of the reaction between ferric H64V SWMb and nitrite. (**A**) UV/visible spectra of 18 µM ferric H64V SWMb (red trace); 80 mM nitrite (black trace), 18 µM ferric H64V SWMb + 80 mM nitrite pre-mixing (blue trace); 18 µM ferric H64V SWMb + 80 mM nitrite post-mixing (green trace). (**B**) Difference spectrum ferric-nitrite H64V SWMb *minus* ferric H64V SWMb at 277 K red trace) and 298 K (blue trace). (**C**) nIR RT-MCD spectrum of ferric H64V SWMb before (765 µM; black trace) and after (383 µM; red trace) the addition of 100 mM nitrite. The green trace is the calculated spectrum of pure ferric nitrite H64V SWMb. (**D**) X-band EPR spectrum of ferric H64V SWMb before (470 µM; black trace) and after (452 µM; red trace) the addition of 100 mM nitrite. The spectrum of the reaction product was simulated (green trace) using three components as described in the methods.

To confirm our interpretation that the features in the RT-MCD spectra of the nitrite bound form of WT SWMb arise from a nitrite ion bound to the ferric heme in the nitrito orientation we examined a variant of SWMb in which the distal histidine (His-64) has been substituted with valine (H64V). The X-ray structure of the nitrite bound form of the H64V variant of HHMb shows that the nitrite ion is bound to the ferric heme in the nitro-orientation (Figure 1B) [16]. Reaction of H64V SWMb with nitrite leads to a shift in the Soret maximum from 395 nm to 415 nm which, because of the overlap in the absorbance spectra of H64V SWMb and nitrite (Figure 4A), is easier to see in the difference spectrum (Figure 4B). Interestingly the amplitude (peak to trough) of the difference spectrum increases as the temperature is reduced from 298 K to 277 K (Figure 4B) indicating the extent of nitrite binding increases as the temperature decreases.

The negligible intensity in the Soret region of the RT-MCD spectrum of untreated H64V SWMb confirms that the heme-iron is high-spin five-coordinate (Supplementary Figure S2B). However, this means that, although nitrite binds sub-stoichiometrically at the protein concentrations needed for MCD, the MCD spectrum of the complex is clearly resolved. A symmetrical bisignate feature centred at ≈420 nm indicates the formation of a new low-spin ferric species.

Before the addition of nitrite, the RT nIR MCD spectrum of the H64V variant is characterised by two features that are typical for five-coordinate high-spin ferric heme; a trough at 657 nm (CT_2_) and a derivative shaped feature at ≈1300 nm (CT_1_) (Figure 4C). Upon addition of 200 mM nitrite, the intensity of the MCD CT_2_ trough at 657 nm diminishes by ≈25%. No additional CT_2_ band is observed and the bisignate Soret feature emerges at ≈420 nm (Figure 4C). These observations suggest that reaction of NO_2_^−^ with a sub-stoichiometric population of the ferric heme yields a species that is low-spin in nature and without a thermally accessible high-spin excited state. At the same time a new nIR-MCD CT_LS_ band appears at ≈1610 nm. In principle, the CT_LS_ spectrum of the bound population can be obtained by subtraction of a sub-stoichiometric portion of the unbound CT_2_ spectrum so as to minimise the latter in the extracted spectrum. However, in practice, the vibrational side-band of the 1610 nm peak overlaps the CT_2_ feature, resulting in a degree of ambiguity in the fraction bound and therefore in the intensity of the CT_LS_. Assuming a bound fraction in the range 0.25–0.35 yields a realistic side-band shape. The dashed green line in Figure 4C shows the calculated spectrum of pure nitrite bound H64V SWMb obtained assuming 0.3 of the heme is bound by nitrite. The maximum at 1610 nm and the intensity are characteristic of ferric heme with bis-nitrogen co-ordination and so consistent with nitrite binding to H64V SWMb in the nitro-mode.

Finally, we recorded the CW X-band EPR spectra of H64V SWMb at 10 K before and after the addition of 100 mM NO_2_^−^ (Figure 4D). Before the addition of nitrite (black trace) the spectrum, as expected, is that of a pure high-spin Ferric heme with a major derivative-shaped g_⊥_ feature at g ≈ 6.0 and a minor feature g_||_ ≈ 2.0. After the addition of nitrite (red trace) the high-spin signal, although still present, represents only ∼1% of the total heme and is replaced by two low-spin rhombic trios with different g_z_ = 3.16 (89%) and g_z_ = 2.93 (10%). Although the almost complete binding of the protein at liquid helium temperatures (Figure 3B) contrasts with the sub-stoichiometric binding at room-temperature it is very much consistent with the strong temperature dependence of nitrite binding to this variant noted above.

### Characterisation of the ferric form of the H93K variant of Sperm Whale myoglobin

**Figure 5.**
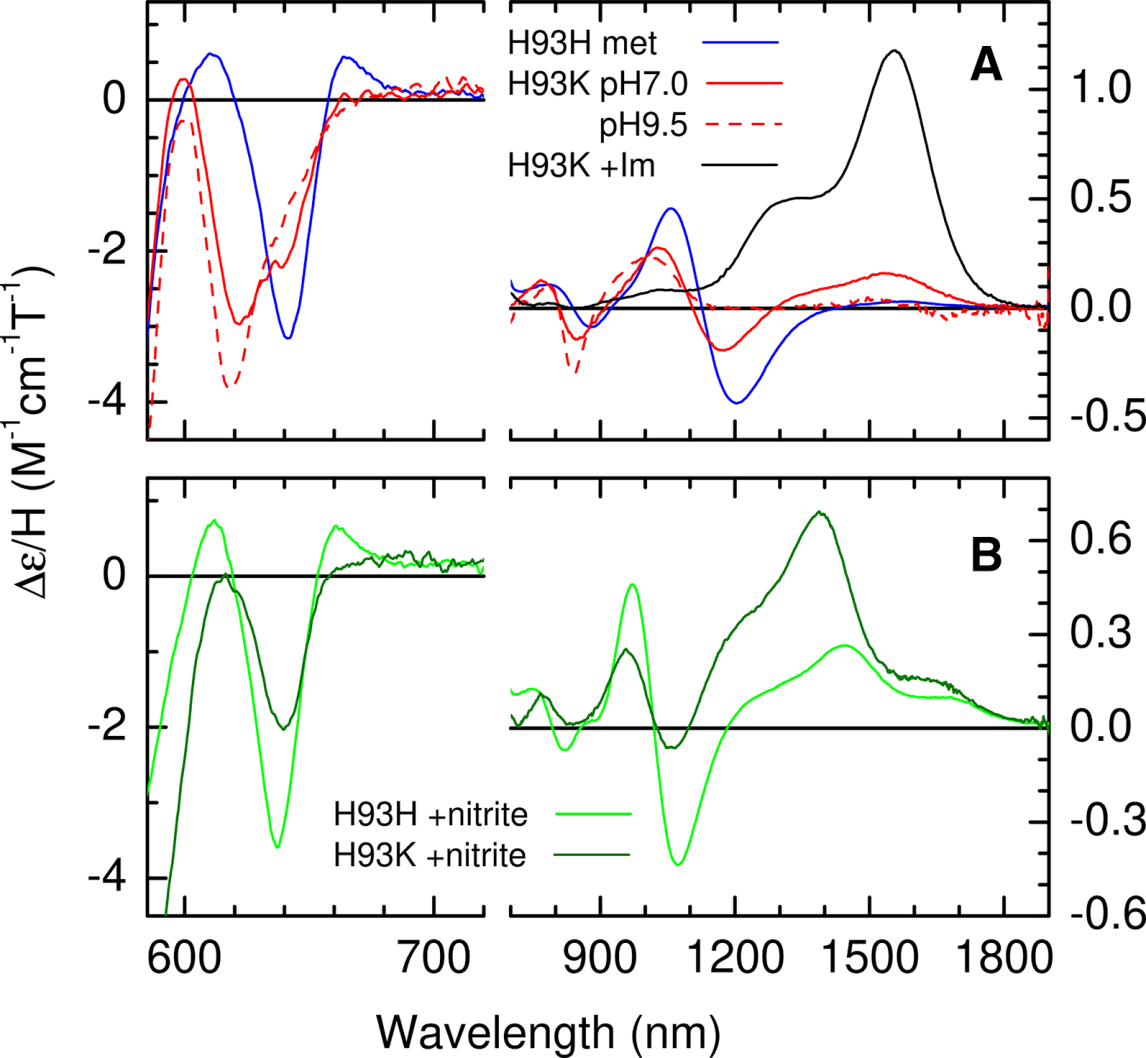
Effects of pH and ligand binding on the nIR MCD Spectra of ferric H93K SWMb. (**A**) nIR MCD spectrum (580–1900 nm) of ferric WT SWMb (790 µM; blue trace) and ferric H93K SWMb at pH 7.0 (470 µM; solid red trace) and pH 9.5 (180 µM; dashed red trace). The product of the reaction of ferric H93K SWMb (490 µM;) with 10 mM imidazole at pH 7.0 is indicated by the black trace. (**B**) nIR MCD spectrum (580–1900 nm) of ferric WT SWMb (640 µM; green trace) and ferric H93K SWMb (428 µM; dark green trace) after reaction with of 100 mM nitrite.

To assess the impact of substituting the proximal histidine with lysine on nitrite binding and reactivity we engineered, expressed and purified the novel H93K variant of SWMb. The presence of the His → Lys substitution in the purified protein was confirmed by mass spectroscopy. We confirmed that the substitution was in the correct position by adding of 100 mM imidazole to a sample of H93K SWMb and recording the electronic absorption spectrum. This showed the anticipated changes which indicate replacement of the distal water ligand by imidazole causing the ferric heme to switch from high-spin to low-spin (Supplementary Figure S3A). Further investigation of the imidazole complex using nIR-MCD revealed a positively signed CT_LS_ band arising from a low-spin Ferric heme with a maximum at 1555 nm (Figure 5A). This is blue-shifted by ≈50 nm compared with the band arising from imidazole/histidine co-ordinated myoglobin [26], but falling in the same region reported for two other histidine/amine coordinated ferric hemes; cytochrome *f* [27] and the amine complex of human haemoglobin [28] (Table 2) and confirms that substitution of histidine 93 with lysine does indeed lead to a change in proximal ligation.

**Table 2:** Characteristic MCD spectroscopic features of ferric hemes with different axial ligand sets

Axial ligands to Ferric Heme	Trough of the high-spin CT_2_ feature in the visible MCD	Cross-over of the high-spin CT_1_ feature in the nIR MCD	Peak of the low-spin CT_LS_ feature in the nIR MCD
His/^−^	Mb (NCBr) 663 nm [29], 665 nm [44] HRP 658 nm [34]	HRP 1200 nm [34]	
His/H_2_O	MB 645 nm [29], 646 nm [30] SWMb 642 nm (this work)	Mb 1066 nm, 1070 nm [31] SWMb 1100 nm (this work), Hb 1105 nm [45]	
His/OH^−^	Mb 615 nm [31], Mb 618 nm [30]. HRP 580 nm [46]	LgHb 805 nm [47] Hb 810 nm [45]	Mb 1000 nm [48], 1040 nm [49], 1050 nm [31]. HRP 1100 nm [34], 1180 nm [50]. LgHb 1015 nm [47]. Hb 1015 nm [45]
His/N_3_^−^	Mb 640 nm [51], Mb 644 nm [30]	Mb 990 nm [31], Mb 948 nm [48] Hb ca 950 nm [45]	Mb 1220 nm [48], 1295 nm [31]. Hb 1275 nm [45]
His/HCOO^−^	Mb 634 nm [52]	Hb 973 nm [28]	
His/H_3_CCOO^−^	Mb 645 nm [34]	Hb 1113 nm [28]	
His/His^−^			Imidazole complexes of Mb 1395 nm [49] and LgHb 1350 nm [47]
His/His			Imidazole complexes of Mb 1600 nm [49], 1530 nm [48] and Soybean LgHb 1350 nm [47]
His/Lys			rape/charnock Cyt *f* 1520 nm [53]. spinach Cyt *f* 1506 nm [27]. alkaline Cyt *c* 1465 nm[33]. Imidazole complex of H93K SWMb 1555 nm (this work)
Lys/H_2_O	H93K SWMb 639 nm (this work)	H93K SWMb 1100 nm (this work)	
Lys/OH^−^	H93K SWMb 622 nm (this work)	H93K SWMb 810 nm (this work)	H93K SWMb 1050 nm (this work)
Lys/Lys			Ferric OEP *bis* (*n*-butylamine) 1320 nm [54]

The UV/vis absorption spectrum of the ferric form of the H93K variant of SWMb at pH 7.0 superficially resembles that of WT SWMb with a Soret maximum at ≈408 nm (Supplementary Figure S3A), but inspection of the visible region reveals clear differences. The RT UV/vis MCD spectrum (Supplementary Figure S3B) shows that these result from the presence of three different forms of the ferric heme. A significant increase in the intensity of the bisignate MCD Soret feature centred at 414 nm (as compared with that of purely high-spin WT SWMb) indicates the presence of a low-spin species. However, overlapping CT_2_ bands in the 600–660 nm region also reveals two high-spin species. Information on the nature of these species is provided by the full nIR MCD spectrum (Figure 5A). The two overlapping CT_2_ bands with troughs at ≈622 nm and near 639 nm are partnered by bisignate CT_1_ bands centred at 810 nm and 1100 nm. The bands are assigned by comparison with the features arising from the equivalent, well documented, species in WT SWMb [29–31]. The CT_2_/CT_1_ band pair at 639/1100 nm is assigned to high-spin ferric heme with Lys/H_2_O coordination and the 622/810 nm pair to the high-spin component of a ferric heme with Lys/HO^−^ coordination.

As noted above, the His/HO^−^ species formed by deprotonation of the distal H_2_O ligand at alkaline pH in WT SWMb is itself in a thermal spin-equilibrium between high- and low-spin states [23,24]. An analogous low-spin component of Lys/HO^−^ in the H93K SWMb variant would account for the increased Soret MCD intensity. This interpretation of the data is supported by the nIR MCD at pH 9.5 where the features at 622/810 nm associated with Lys/ HO^−^ have increased at the expense of those from Lys/H_2_O at 639/1100 nm. A positively signed CT_LS_ band, assigned to the low-spin component of Lys/HO^−^ coordinated ferric heme has also increased and is now resolved at 1010 nm. This is blue-shifted by ∼20 nm compared with the equivalent band in His/HO^−^ WT SWMb [32]. A weak CT_LS_ band observed at 1510 nm in the pH 7.0 sample would be consistent with some degree of ligation by the distal pocket histidine giving rise to low-spin Lys/His coordinated Ferric heme [33], possibly reflecting an inherent instability in the heme pocket of this variant. Taking the pH below 7.0 causes the protein to become unstable and denature thus imposing a lower limit on the accessible range.

**Figure 6.**
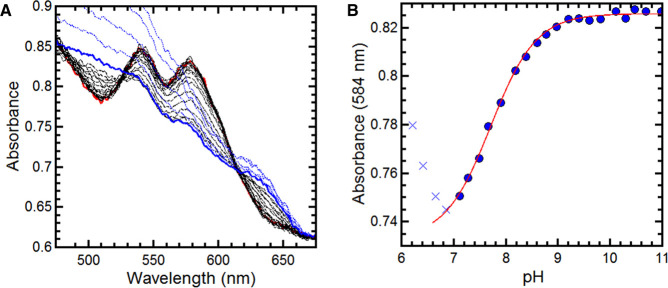
Changes in the UV-visible spectrum of ferric H93K SWMb as a function of pH. (**A**) Changes in the visible region of the spectrum (490–680 nm) of ferric H93K SWMb over the range pH = 11 (red trace) to pH = 7.2 (blue trace) (**B**) Dependence of absorbance (584 nm) of H93K SWMb on pH. Solid blue circles of represent the absorbance at 584 nm determined from the spectra shown in **A**. Blue crosses represent the absorbance at 584 nm associated with denatured protein. The red trace is a fit to a single ionising species of pK_a_ = 7.78. All experiments were carried out in a mixed buffer system as described in Materials and Methods.

The pH at which deprotonation of water bound to the distal face of ferric heme occurs is known to vary according to the nature of the heme pocket [25,34]. The observation of a mixture of H_2_O and HO^−^ distal ligands in H93K at *neutral* pH would indicate that the substituted proximal ligand is exerting a significant influence on the properties of the distal water ligand. In WT SWMb this alkaline transition occurs with a p*K*_a_ of 8.9 and the heme is purely high-spin His/H_2_O at pH 7.0. A pH titration of H93K was monitored by UV-visible absorbance spectroscopy (Figure 6). At pH 9.5, there are defined maxima at 540 and 577 nm. By plotting the change in absorbance at 583 nm as a function of pH, a p*K*_a_ of 7.8 was determined for the alkaline transition in H93K, significantly lower than that reported for WT SWMb [35] (Table 1). At pH7.0, close to this lowered p*K*_a_ value, a Lys/OH^−^ to Lys/H_2_O ratio of ≈15 : 85 would be anticipated, consistent with the mixed species observed in the MCD spectra.

### Reaction of the ferric form of the H93K variant of Sperm Whale myoglobin with nitrite and spectroscopic characterisation of the product

The amplitudes of the reaction progress curves measured in the stopped-flow spectrometer show a similar hyperbolic dependence on [NO_2_^−^] to that shown by WT SWMb (Supplementary Figure S4) with the following estimated dissociation constant; *K*_d_ = 1.92 mM (H93K) (Table 1).

Addition of 100 mM nitrite to ferric H93K SWMb at pH 7.0 causes shifts in the Soret and αβ (visible) absorption bands that are similar to those observed for nitrite addition to WT (Supplementary Figure S5A), but the MCD at UV-visible wavelengths shows there to be quantitative differences between the two nitrite treated proteins (Supplementary Figure S5B). The increase in Soret and αβ band intensity is greater for H93K than for WT indicating a higher proportion of low-spin formation, but a high-spin band is still also observed at ≈640 nm. A detailed comparison can be made via the nIR MCD shown in Figure 5B. The high-spin CT_2_/CT_1_ bands at 637/1020 nm observed for WT-NO_2_^−^ are observed at similar wavelengths (640/1005 nm) for H93K, again suggesting nitrito- coordination, but the intensities are more than halved. The low-spin CT band appears at 1390 nm for H93K-NO_2_^−^ and, in contrast, is several times more intense than the equivalent WT-NO_2_ CT band at 1445 nm. These data are summarised in Table 3. If it assumed that the high-spin CT_2_ feature and the low-spin CT_LS_ band are of comparable intensities for the two species, then the low-spin content can be estimated as 24% and 64% for WT-NO_2_^−^ and H93K-NO_2_^−^, respectively.

**Table 3:** Summary of the RT-MCD properties of ferric SWMb nitrite complexes used in this study

SWMb Variant	Trough of the high-spin CT_2_ feature in the visible MCD	Cross-over of the high-spin CT_1_ feature in the nIR MCD	Peak of the low-spin CT_LS_ feature in the nIR MCD
WT	637 nm	1020 nm	1444 nm
H93K	640 nm	1005 nm	1390 nm
H64V			1610 nm

### Reaction of the ferrous forms of WT and H93K SWMb with nitrite

**Figure 7.**
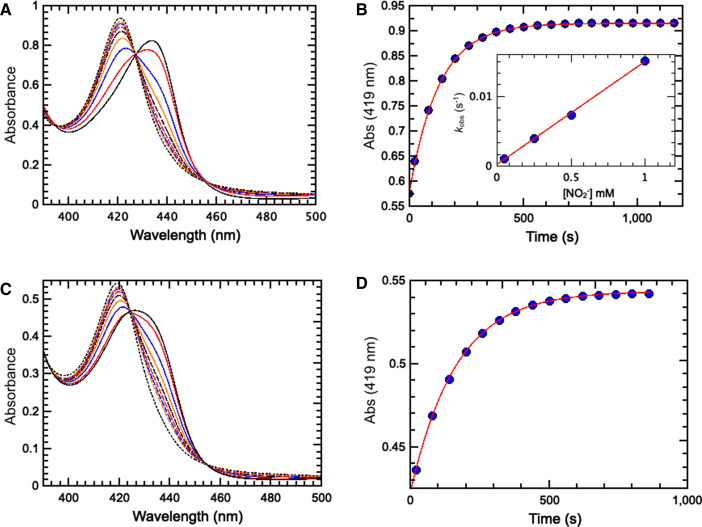
Reaction of the ferrous forms of WT and H93K SWMb with nitrite. (**A**) Time resolved UV/vis spectra observed in the reaction of dithionite reduced WT SWMb with 0.5 mM nitrite. The first spectrum (solid black trace) was recorded after 36 s with subsequent spectra recorded at 60 s intervals. (**B**) Progress of the reaction of dithionite reduced WT SWMb with 0.5 mM nitrite monitored at 419 nm. Blue circles are data points derived from the spectra shown in **A** and the red line is a fit to single exponential (*k*_obs_ = 7.7 × 10^−3^ s^−1^). **Inset**: Dependence of *k*_obs_ on nitrite. (**C**) Time resolved UV/vis spectra observed in the reaction of dithionite reduced H93K SWMb with 50 µM nitrite. The first spectrum (solid black trace) was recorded after 36 s with subsequent spectra recorded at 60 s intervals. (**D**) Progress of the reaction of dithionite reduced H93K SWMb with 50 µM nitrite monitored at 419 nm. Blue circles are data points derived from the spectra shown in **C** and the red line is a fit to single exponential (*k*_obs_ = 6.05 × 10^−3^ s^−1^).

The rate of nitrite reduction by ferrous WT SWMb was estimated using a previously described method [20] that exploits the fact that the rate of (re)reduction in myoglobin by 30 mM dithionite *k*_obs_ = 30 s^−1^ [36] is much faster than the rate of nitrite reduction. Addition of 0.5 mM nitrite to reduced WT SWMb under these condition leads to a rapid decrease in absorbance at 434 nm and a concomitant increase in absorbance at 419 nm (Figure 7A). The shift in the Soret maximum is consistent with the formation of a ferrous-nitrosyl species. The progress of the reaction monitored at 419 nm is described in terms of a first order rate constant *k*_obs_ = 7.7 × 10^−3^ s^−1^ (Figure 7B) that is linearly dependent upon [nitrite] between 0.05 mM and 1 mM (Figure 7B inset) and yields a bimolecular rate constant *k*_+1_ of 14.8 M^−1^ s^−1^ (Table 1).

Since the H93K variant of SWMb has not been described previously we recorded the UV-visible absorption spectra of the nitrosyl complexes of the protein in both ferric and ferrous states (Supplementary Table S1). The close resemblance of the Fe(II) and Fe(II)-NO forms of the H93K variant to the equivalent forms of WT SWMb, together with a similar rate of (re)reduction by 30 mM dithionite allows the rate of nitrite reduction to be estimated using the same method except that the reaction is initiated with 50 µM nitrite in order to record the entire progress of the reaction (Figure 7C,D). The ≈7-fold rate enhancement observed in the H93K mutant (Table 1) suggests a role for lysine in facilitating nitrite reduction. Since NO_2_^−^ binding and reduction takes place at the distal face of the heme it appears that the increased rate of nitrite reduction arises from the change in the environment of the distal pocket caused by substitution of the proximal ligand that is described above.

## Discussion

Here we establish that WT SWMb reacts rapidly with free nitrous acid to form a stable product that has a RT-MCD spectrum containing signals that arise from both high- and low-spin hemes. This is in keeping with an earlier study of nitrite binding to ferric HHMb in solution which also found evidence for the presence of both high- and low-spin forms of the heme at ambient temperatures based on changes in the UV/vis spectrum [37]. Low-temperature EPR spectroscopy of the same sample revealed a low-spin species which, based on the g = 3 signal, the authors attributed to the presence of a nitrogenous ligand; i.e. nitrite binding in the nitro-orientation and accounting for 44% of the total heme [37]. The remainder of heme was reported to be a high-spin species that originates from nitrito-bound heme, rather than a proportion of the protein that retained unreacted His/H_2_O ligated ferric heme. This led the authors to the conclusion that both nitro- and nitrito- species were present in their samples [37].

The present study clearly shows that both spin-states originate from a thermal equilibrium of high- and low-spin forms of individual species in which the low-spin is the ground state; an observation that is consistent with earlier magnetic susceptibility studies [24]. The wavelengths of the two high-spin ferric CT bands in the MCD spectrum are characteristic of coordination by a RO^-^ ligand and are consistent with nitrito- coordination. The nIR CT band (CT_LS_ band) that is associated with the low-spin component of the thermal spin mixture, lies at an unusual wavelength (1444 nm) that is outside the range observed for distal bis-nitrogen coordination e.g. from lysine or histidine providing further evidence of nitrito-coordination. This interpretation is broadly in accordance with a resonance Raman study of the nitrite bound form of WT HHMb at ambient temperature which only identified a high-spin nitrito-bound species [38], with no evidence of resonances associated with the nitro-species characterised in a variant of cytochrome *c*′ [39].

The EPR spectrum of the reaction product recorded at 10 K is dominated by signals arising from low-spin ferric heme with properties similar to those previously reported for nitrite treated HHMb [37] and human haemoglobin and attributed to His/nitro-ligated heme [37,40]. However, this assignment was made on the basis of *g*-value analysis using methods that can be ambiguous in that different ligand sets give rise to comparable EPR spectra depending on their relative orientation. DFT based simulations of nitrito-ligated heme, in which the ligand is orientated by H-bonding to the distal histidine, was able to account for the experimentally determined EPR *g*-values [41]. However, it is important to note that because these calculations are performed using a model in which the nitrite orientation is derived from that in the crystal structures, the resulting EPR *g*-values are characteristic of that particular conformation, but would not necessarily be diagnostic of nitrito-binding in other systems in which the two axial ligands adopt different relative orientations.

The asymmetry of the g ≈ 3.0 feature in our samples is indicative of some degree of heterogeneity, possibly due to the presence of both *cis* and *trans* linkage isomers of the nitrito-bound species [39]. This is accounted for in our simulation of the experimental data (Figure 3B) by including more than one low-spin species and together these represent ≈98% of the heme iron the remaining 2% being accounted for by the high-spin nitrito-species. These relative proportions of high- and low-spin forms at 10 K are consistent with a thermal equilibrium with a low-spin ground state. Unfortunately, MCD spectroscopy cannot distinguish between the *cis* and *trans* isomers of nitrito-bound ferric heme, we plan to resolve these in the future using advanced EPR methodologies in combination with ^15^N-labelled nitrite.

The question then arises as to the spectroscopic properties of an authentic nitro-coordinated ferric heme. The X-ray crystallographic structures of the nitrite adduct of oxidised WT HHMb show that the nitrite ion is bound to the ferric heme iron in the nitrito-conformation just as we observe in our solution studies. Since the X-ray structure of the nitrite complex of HHMb H64V shows nitrite bound to the ferric heme via the nitrogen (Figure 1B) [16], it is reasonable to assume that the same species exists in solution. Therefore, we used the same combination of low-temperature EPR and RT-MCD to characterise the minority species formed following the addition of 100 mM nitrite to SWMb H64V. This species gives rise to a CT_LS_ band in the nIR MCD spectrum at ≈1600 nm, similar to a number of other bis-nitrogen coordinated hemes (Table 2) and hence is assigned to nitro-coordination. However, the sample is still predominantly high-spin, but this because of the high proportion of unbound five-coordinate starting material resulting from a greatly increased *K*_d_ for nitrite binding. Interestingly the extent of nitrite binding to SWMb H64V is strongly temperature dependent and as a consequence the EPR at 10 K of SWMb H64V treated with nitrite suggests near-complete binding that is indicated by two low-spin ferric species with slightly different *g*-values. These *g*-values are not the same as those of the nitrito-derivative found in WT SWMb WT protein. Moreover, the value of the *g*_z_ component for both these nitro-bound forms is higher than that predicted for the nitrito-bound form by DFT analysis [41].

These data along with the X-ray studies are consistent with the proposal that the distal histidine in WT SWMb orientates the nitrite ligand in the nitrito-orientation through hydrogen bonding. Substitution with valine removes this possibility and allows the ligand to bind in the nitro-orientation. Having reliable spectroscopic fingerprints of NO_2_^-^ bound ferric heme in both nitrito- and nitro-orientations will ultimately allow the re-evaluation of spectroscopic signals associated with nitrite binding to more complex heme proteins including NrfA [3]. In the short term, it allowed us to explore the consequences of substitution of the proximal heme ligand of WT SWMb, Histidine-93, with lysine the residue that serves as the proximal heme ligand in the bacterial nitrite reductase NrfA. We were particularly interested in whether the proximal ligand exerted any influence on nitrite binding and/or reactivity.

Replacement of the proximal histidine with lysine yields a variant SWMb that in the ferric state has properties that are qualitatively similar to those of WT SWMb, but which exhibits quantitative differences with respect to its reaction with nitrite. As is the case for WT SWMb, we observe nitrito-binding and a thermal spin-equilibrium with a low-spin ground state, but the MCD CT bands associated with both high- and low-spin forms of the nitrito species are shifted to slightly shorter wavelengths, behaviour that has been observed in several six-coordinate low-spin heme systems which contain a proximal lysine residue (Table 2).

In the case of H64K SWMb the proportion of low-spin heme in the thermal mixture is 64% rather than the 25% found in WT SWMb. This difference can be explained as follows. If the energy separation, ΔE, between the high-spin and low-spin states, in either order, is very small compared with *k*_T_ at room temperature then the population will be spread across the two states according to their degeneracies (2S + 1) and a sample 25% in low-spin would be anticipated in the RT-MCD. This was observed for the NO_2_^−^ bound species and it therefore required a low-temperature EPR measurement to identify the ground state as low-spin. A low-spin component far greater than 25% is observed for H93K-NO_2_^−^ indicating a low-spin ground state with a significantly larger Δ E such that the high-spin state is not fully populated at room-temperature. On the basis of the similar CT band positions we conclude that both WT and H93K are bound in the nitrito- conformation and that, therefore, the increase in ligand strength leading to the different spin-mixtures is a result of the substitution of histidine with lysine.

Unexpectedly, substitution of the proximal histidine with lysine also appears to cause a change in the environment of the distal pocket which is observed through a lowering of the p*K*_a_ of the water bound to the iron in the heme from p*K*_a_ = 8.9 (WT SWMb) to p*K*_a_ = 7.8 (H93K). This change in the distal pocket environment may also account for the lower *K*_d_ for nitrite binding relative to wild-type by stabilising the nitrito-bound form accounting for the lower dissociation rate constant.

The final question concerns whether nitrito-orientation of substrate to the ferric heme is associated with rapid nitrite reduction by ferrous SWMbs. The bimolecular rate constant (17.8 M^−1^ s^−1^) that we observe for the reaction of ferrous WT SWMb with nitrite is somewhat faster than those previously reported by Tiso et al. [42] and Wu et al. [43]; 5.6 ± 0.6 M^−1^ s^−1^ and 6.1 ± 0.4 M^−1^ s^−1^, respectively. This is accounted for by the fact that our measurements were made at pH 7.0 rather than pH 7.4 and as a consequence the concentration of HNO_2_ would be *ca* 2.5-fold higher. Substitution of the distal histidine with valine leads to substantial decrease in the rate of nitrite reduction by ferrous SWMb (Table 1). Tiso et al. [42] reported that the rate of reduction in nitrite by the ferrous form of the H64L variant of SWMb was less than 10-fold than WT SWMb. Could it be that when the distal histidine is replaced by a hydrophobic residue there is a reduced rate of nitrite reduction because the substrate binds in same nitro-orientation observed spectroscopically in the ferric form of H64V SWMb (Figure 4)? The ferric form of H64V variant of HHMb also binds nitrite in the nitro-orientation, and there is a >10-fold reduction in the rate of reduction in nitrite by the ferrous form compared with wild-type [16]. However activity is partially restored to this variant by substituting Val-67 with an arginine residue; the ferrous form of H64V/V67R HHMb having 33% activity relative to WT HHMb. Moreover, binding of nitrite in the nitrito-orientation is also restored in the ferric form of H64V/V67R HHMb [16].

Substitution of His-64 with alanine in SWMb reduces the nitrite reductase activity *ca* 3-fold. However, when this is combined with a second substitution, in which Phe-43 is replaced with histidine the ferrous form of the resultant F43H/H64A variant reduces nitrite *ca* 8-fold faster than wild-type [43]. Again coordination of nitrite binding to the ferric heme of F43H/H64A SWMb is the nitrito-orientation, although it is indirect; the ion is coordinated between the imidazole group of His-43 and the water (W2) that serves as the distal ligand to the heme iron [43]. Substitution of His-64 with alanine creates a channel that leads to the heme distal pocket that contains seven interconnected water molecules (W1–W7). Therefore, it appears that that rapid nitrite reduction by ferrous myoglobins is associated with a residue at one of positions 43, 64 or 67 that can coordinate the substrate to the ferric heme iron in the nitrito orientation, and one or more water molecules with an appropriate p*K*_a_ to provide protons for the reaction.

We propose that in the case of the H93K SWMb the ca 8-fold enhancement in the rate of nitrite reduction by the ferrous protein that we report here is not simply due to the change of proximal ligand altering the reactivity of the heme. Instead, it is explained in terms of substitution of the proximal histidine with lysine causing a change in the environment of the distal pocket that facilitates the availability of substrate protons to a nitrito-bound ferrous heme. The changed environment is reported through a lowering of the p*K*_a_ of the water bound to the iron in the ferric heme from p*K*_a_ = 8.9 (WT SWMb) to p*K*_a_ = 7.8 (H93K SWMb). This argument assumes that nitrite binding to ferrous heme is in the same orientation that observed spectroscopically in the ferric state. Whilst the rapid transformation of nitrite to nitric oxide by reduced myoglobin at ambient temperatures means that this is difficult to test experimentally, we note that at cryogenic temperatures crystals of stable adducts of nitrite with WT HHMb retain the nitrito-conformation after photo-reduction in the heme in an X-ray beam [14]. Work is ongoing in our laboratory to obtain a high-resolution X-ray structure of ferric H93K SWMb in the presence and absence of bound nitrite that will allow us to understand how any changes in the network of water molecules in the distal pocket that result from the change in proximal ligation might interact with the bound substrate to promote nitrite reduction.

## Open Access

Open access for this article was enabled by the participation of University of East Anglia in an all-inclusive *Read & Publish* pilot with Portland Press and the Biochemical Society under a transformative agreement with JISC.

## Abbreviations

CT: Charge Transfer
CW: Continuous Wave
EPR: Electron Paramagnetic Resonance
Hb: Haemoglobin
HHMb: Horse Heart Myoglobin
Lgb: Leghaemoglobin
Mb: Myoglobin
MCD: Magnetic Circular Dichroism
SWMb: Sperm Whale Myoglobin

## References

[1] Bamford, V.A., Angove, H.C., Seward, H.E., Thomson, A.J., Cole, J.A., Butt, J.N. et al. (2002) Structure and spectroscopy of the periplasmic cytochrome c nitrite reductase from *Escherichia coli*. Biochemistry 41, 2921–2931 10.1021/bi015765d 11863430

[2] Clarke, T.A., Kemp, G.L., Van Wonderen, J.H., Doyle, R.M., Cole, J.A., Tovell, N. et al. (2008) Role of a conserved glutamine residue in tuning the catalytic activity of *Escherichia coli* cytochrome c nitrite reductase. Biochemistry 47, 3789–3799 10.1021/bi702175w 18311941

[3] Lockwood, C.W., Burlat, B., Cheesman, M.R., Kern, M., Simon, J., Clarke, T.A. et al. (2015) Resolution of key roles for the distal pocket histidine in cytochrome C nitrite reductases. J. Am. Chem. Soc. 137, 3059–3068 10.1021/ja512941j 25658043

[4] van Wonderen, J.H., Burlat, B., Richardson, D.J., Cheesman, M.R. and Butt, J.N. (2008) The nitric oxide reductase activity of cytochrome c nitrite reductase from *Escherichia coli*. J. Biol. Chem. 283, 9587–9594 10.1074/jbc.M709090200 18245085

[5] Einsle, O., Messerschmidt, A., Stach, P., Bourenkov, G.P., Bartunik, H.D., Huber, R. et al. (1999) Structure of cytochrome c nitrite reductase. Nature 400, 476–480 10.1038/22802 10440380

[6] Einsle, O., Stach, P., Messerschmidt, A., Simon, J., Kröger, A., Huber, R. et al. (2000) Cytochrome c nitrite reductase from Wolinella succinogenes. Structure at 1.6 A resolution, inhibitor binding, and heme-packing motifs. J. Biol. Chem. 275, 39608–39616 10.1074/jbc.M006188200 10984487

[7] Cunha, C.A., Macieira, S., Dias, J.M., Almeida, G., Goncalves, L.L., Costa, C. et al. (2003) Cytochrome c nitrite reductase from *Desulfovibrio desulfuricans* ATCC 27774. The relevance of the two calcium sites in the structure of the catalytic subunit (NrfA). J. Biol. Chem. 278, 17455–17465 10.1074/jbc.M211777200 12618432

[8] Rodrigues, M.L., Oliveira, T.F., Pereira, I.A. and Archer, M. (2006) X-ray structure of the membrane-bound cytochrome c quinol dehydrogenase NrfH reveals novel haem coordination. EMBO J. 25, 5951–5960 10.1038/sj.emboj.7601439 17139260 PMC1698886

[9] Einsle, O., Messerschmidt, A., Huber, R., Kroneck, P.M. and Neese, F. (2002) Mechanism of the six-electron reduction of nitrite to ammonia by cytochrome c nitrite reductase. J. Am. Chem. Soc. 124, 11737–11745 10.1021/ja0206487 12296741

[10] Wittenberg, B.A. and Wittenberg, J.B. (1989) Transport of oxygen in muscle. Annu. Rev. Physiol. 51, 857–878 10.1146/annurev.ph.51.030189.004233 2653210

[11] Shiva, S., Huang, Z., Grubina, R., Sun, J., Ringwood, L.A., MacArthur, P.H. et al. (2007) Deoxymyoglobin is a nitrite reductase that generates nitric oxide and regulates mitochondrial respiration. Circ. Res. 100, 654–661 10.1161/01.RES.0000260171.52224.6b 17293481

[12] Lundberg, J.O., Weitzberg, E. and Gladwin, M.T. (2008) The nitrate-nitrite-nitric oxide pathway in physiology and therapeutics. Nat. Rev. Drug Discov. 7, 156–167 10.1038/nrd2466 18167491

[13] Copeland, D.M., Soares, A.S., West, A.H. and Richter-Addo, G.B. (2006) Crystal structures of the nitrite and nitric oxide complexes of horse heart myoglobin. J. Inorg. Biochem. 100, 1413–1425 10.1016/j.jinorgbio.2006.04.011 16777231

[14] Yi, J., Orville, A.M., Skinner, J.M., Skinner, M.J. and Richter-Addo, G.B. (2010) Synchrotron X-ray-induced photoreduction of ferric myoglobin nitrite crystals gives the ferrous derivative with retention of the O-bonded nitrite ligand. Biochemistry 49, 5969–5971 10.1021/bi100801g 20568729 PMC2916933

[15] Yi, J., Safo, M.K. and Richter-Addo, G.B. (2008) The nitrite anion binds to human hemoglobin via the uncommon O-nitrito mode. Biochemistry 47, 8247–8249 10.1021/bi801015c 18630930

[16] Yi, J., Heinecke, J., Tan, H., Ford, P.C. and Richter-Addo, G.B. (2009) The distal pocket histidine residue in horse heart myoglobin directs the O-binding mode of nitrite to the heme iron. J. Am. Chem. Soc. 131, 18119–18128 10.1021/ja904726q 19924902 PMC2824769

[17] Springer, B.A. and Sligar, S.G. (1987) High-level expression of sperm whale myoglobin in *Escherichia coli*. Proc. Natl Acad. Sci. U.S.A. 84, 8961–8965 10.1073/pnas.84.24.8961 3321062 PMC299671

[18] Brittain, T., Little, R.H., Greenwood, C. and Watmough, N.J. (1996) The reaction of *Escherichia coli* cytochrome bo with H2O2: evidence for the formation of an oxyferryl species by two distinct routes. FEBS Lett. 399, 21–25 10.1016/S0014-5793(96)01253-7 8980111

[19] Thomson, A.J., Cheesman, M.R. and George, S.J. (1993) Variable-temperature magnetic circular-dichroism. Methods Enzymol. 226, 199–232 10.1016/0076-6879(93)26011-w 8277866

[20] Tejero, J., Sparacino-Watkins, C.E., Ragireddy, V., Frizzell, S. and Gladwin, M.T. (2015) Exploring the mechanisms of the reductase activity of neuroglobin by site-directed mutagenesis of the heme distal pocket. Biochemistry 54, 722–733 10.1021/bi501196k 25554946 PMC4410703

[21] Yi, J. and Richter-Addo, G.B. (2012) Unveiling the three-dimensional structure of the green pigment of nitrite-cured meat. Chem. Commun. 48, 4172–4174 10.1039/c2cc31065a 22430128

[22] Wanat, A., Gdula-Argasinska, J., Rutkowska-Zbik, D., Witko, M., Stochel, G. and van Eldik, R. (2002) Nitrite binding to metmyoglobin and methemoglobin in comparison to nitric oxide binding. J. Biol. Inorg. Chem. 7, 165–176 10.1007/s007750100284 11862553

[23] Beetlestone, J. and George, P. (1964) A magnetochemical study of equilibria between high and low spin states of metmyoglobin complexes. Biochemistry 3, 707–714 10.1021/bi00893a019 14193642

[24] Smith, D.W. and Williams, R.J. (1968) Analysis of the visible spectra of some sperm-whale ferrimyoglobin derivatives. Biochem. J. 110, 297–301 10.1042/bj1100297 5726207 PMC1187209

[25] Gurd, F.R., Falk, K.E., Malmström, B.G. and Vänngård, T. (1967) A magnetic resonance study of sperm whale ferrimyoglobin and its complex with 1 cupric ion. J. Biol. Chem. 242, 5724–5730 10.1016/S0021-9258(18)99360-9 5634191

[26] Gadsby, P.M.A. and Thomson, A.J. (1990) Assignment of the axial ligands of ferric ion in low-spin hemoproteins by near-infrared magnetic circular-dichroism and electron-paramagnetic resonance spectroscopy. J. Am. Chem. Soc. 112, 5003–5011 10.1021/ja00169a002

[27] Simpkin, D., Palmer, G., Devlin, F.J., McKenna, M.C., Jensen, G.M. and Stephens, P.J. (1989) The axial ligands of heme in cytochromes: a near-infrared magnetic circular dichroism study of yeast cytochromes c, c1, and b and spinach cytochrome f. Biochemistry 28, 8033–8039 10.1021/bi00446a010 2557894

[28] Rawlings, J., Stephens, P.J., Nafie, L.A. and Kamen, M.D. (1977) Near-infrared magnetic circular-dichroism of cytochrome c′. Biochemistry 16, 1725–1729 10.1021/bi00627a032 192272

[29] Bracete, A.M., Sono, M. and Dawson, J.H. (1991) Effects of cyanogen-bromide modification of the distal histidine on the spectroscopic and ligand-binding properties of myoglobin: magnetic circular-dichroism spectroscopy as a probe of distal water ligation in ferric high-spin histidine-bound heme-proteins. Biochim. Biophys. Acta 1080, 264–270 10.1016/0167-4838(91)90012-O 1954234

[30] Vickery, L., Nozawa, T. and Sauer, K. (1976) Magnetic circular-dichroism studies of myoglobin complexes: correlations with heme spin state and axial ligation. J. Am. Chem. Soc. 98, 343–350 10.1021/ja00418a005 173751

[31] Eglinton, D.G., Gadsby, P.M.A., Sievers, G., Peterson, J. and Thomson, A.J. (1983) A comparative-study of the low-temperature magnetic circular-dichroism spectra of horse heart metmyoglobin and bovine liver catalase derivatives. Biochim. Biophys. Acta 742, 648–658 10.1016/0167-4838(83)90284-4 6838894

[32] Cheesman, M.R., Greenwood, C. and Thomson, A.J. (1991) Magnetic circular-dichroism of hemoproteins. Adv. Inorg. Chem. 36, 201–255 10.1016/S0898-8838(08)60040-9

[33] Gadsby, P.M., Peterson, J., Foote, N., Greenwood, C. and Thomson, A.J. (1987) Identification of the ligand-exchange process in the alkaline transition of horse heart cytochrome c. Biochem. J. 246, 43–54 10.1042/bj2460043 2823795 PMC1148238

[34] Kobayashi, N., Nozawa, T. and Hatano, M. (1977) Magnetic circular-dichroism studies on acid and alkaline forms of horseradish-peroxidase. Biochim. Biophys. Acta 493, 340–351 10.1016/0005-2795(77)90190-8 19085

[35] Brunori, M., Amiconi, G., Antonin, E., Wyman, J., Zito, R. and Fanelli, A.R. (1968) The transition between ‘acid’ and ‘alkaline’ ferric heme proteins. Biochim. Biophys. Acta 154, 315–322 10.1016/0005-2795(68)90045-7 5637052

[36] Cox, R.P. and Hollaway, M.R. (1977) The reduction by dithionite of Fe(III) myoglobin derivatives with different ligands attached to the iron atom. A study by rapid-wavelength-scanning stopped-flow spectrophotometry. Eur. J. Biochem. 74, 575–587 10.1111/j.1432-1033.1977.tb11427.x 856586

[37] Silaghi-Dumitrescu, R., Svistunenko, D.A., Cioloboc, D., Bischin, C., Scurtu, F. and Cooper, C.E. (2014) Nitrite binding to globins: linkage isomerism, EPR silence and reductive chemistry. Nitric Oxide 42, 32–39 10.1016/j.niox.2014.08.007 25172022 PMC4256065

[38] Lambrou, A., Ioannou, A. and Pinakoulaki, E. (2016) Spin crossover in nitrito-myoglobin as revealed by resonance raman spectroscopy. Chemistry 22, 12176–12180 10.1002/chem.201601738 27417111

[39] Nilsson, Z.N., Mandella, B.L., Sen, K., Kekilli, D., Hough, M.A., Moenne-Loccoz, P. et al. (2017) Distinguishing nitro vs nitrito coordination in cytochrome c′ using vibrational spectroscopy and density functional theory. Inorg. Chem. 56, 13205–13213 10.1021/acs.inorgchem.7b01945 29053273 PMC5677563

[40] Schwab, D.E., Stamler, J.S. and Singel, D.J. (2010) EPR spectroscopy of nitrite complexes of methemoglobin. Inorg. Chem. 49, 6330–6337 10.1021/ic902085s 20666390 PMC4498954

[41] Sundararajan, M. and Neese, F. (2015) Distal histidine modulates the unusual o-binding of nitrite to myoglobin: evidence from the quantum chemical analysis of EPR parameters. Inorg. Chem. 54, 7209–7217 10.1021/acs.inorgchem.5b00557 26172912

[42] Tiso, M., Tejero, J., Basu, S., Azarov, I., Wang, X., Simplaceanu, V. et al. (2011) Human neuroglobin functions as a redox-regulated nitrite reductase. J. Biol. Chem. 286, 18277–18289 10.1074/jbc.M110.159541 21296891 PMC3093900

[43] Wu, L.B., Yuan, H., Gao, S.Q., You, Y., Nie, C.M., Wen, G.B. et al. (2016) Regulating the nitrite reductase activity of myoglobin by redesigning the heme active center. Nitric Oxide 57, 21–29 10.1016/j.niox.2016.04.007 27108710

[44] Matsuoka, A., Kobayashi, N. and Shikama, K. (1992) The soret magnetic circular-dichroism of ferric high-spin myoglobins: a probe for the distal histidine residue. Eur. J. Biochem. 210, 337–341 10.1111/j.1432-1033.1992.tb17426.x 1446682

[45] Stephens, P., Sutherland, J., Cheng, J. and Eaton, W. (1976) The study of spin-states of heme proteins by near infrared magnetic circular dichroism. In Excited States of Biological Molecular Processes (Birks, J., ed.), p. 434, Wiley, Chichester

[46] Nozawa, T., Kobayashi, N. and Hatano, M. (1976) Magnetic circular-dichroism studies on horseradish-peroxidase. Biochim. Biophys. Acta 427, 652–662 10.1016/0005-2795(76)90209-9 1268223

[47] Sievers, G., Gadsby, P.M.A., Peterson, J. and Thomson, A.J. (1983) Magnetic circular-dichroism spectra of soybean leghemoglobin-a at room-temperature and 4.2-K. Biochim. Biophys. Acta 742, 637–647 10.1016/0167-4838(83)90283-2 6838894

[48] Nozawa, T., Yamamoto, T. and Hatano, M. (1976) Infrared magnetic circular-dichroism of myoglobin derivatives. Biochim. Biophys. Acta 427, 28–37 10.1016/0005-2795(76)90282-8 1260002

[49] Gadsby, P.M.A. and Thomson, A.J. (1982) Identification of the imidazolate anion as a ligand in metmyoglobin by near-infrared magnetic circular-dichroism spectroscopy. FEBS Lett. 150, 59–63 10.1016/0014-5793(82)81304-5

[50] Foote, N., Gadsby, P.M.A., Berry, M.J., Greenwood, C. and Thomson, A.J. (1987) The formation of ferric heme during low-temperature photolysis of horseradish-peroxidase compound-I. Biochem. J. 246, 659–668 10.1042/bj2460659 2825645 PMC1148330

[51] Springall, J. (1977) Low Temperature Magnetic Circular Dichroism Studies of Some Haemoproteins, University of East Anglia

[52] Dawson, J. and Dooley, D. (1989) Magnetic circular dichroism spectroscopy. In Physical Bioinorganic Chemistry, Iron Porphyrins Part III (Lever, A. and Gray, H., eds), p. 3, VCH Publishers Inc, New York

[53] Rigby, S.E.J., Moore, G.R., Gray, J.C., Gadsby, P.M.A., George, S.J. and Thomson, A.J. (1988) Nmr, electron-paramagnetic-Res and magnetic-Cd studies of cytochrome-F: identity of the heme axial ligands. Biochem. J. 256, 571–577 10.1042/bj2560571 3223931 PMC1135448

[54] Gadsby, P.M.A. and Thomson, A.J. (1986) Low-temperature electron-paramagnetic-Res and near-infrared mcd studies of highly anisotropic low-spin ferriheme complexes. FEBS Lett. 197, 253–257 10.1016/0014-5793(86)80337-4

