# Supplementary material for: Influence of the heme distal pocket on nitrite binding orientation and reactivity in Sperm Whale myoglobin
Source: Biochem J. 2021 Feb 26;478(4):927–42. doi: 10.1042/BCJ20200596 (PMC7925009; doi:10.1042/BCJ20200596)
Supplement: Supplementary Figures S1-S5 and Table S1 [file BCJ-478-927-s1.pdf]

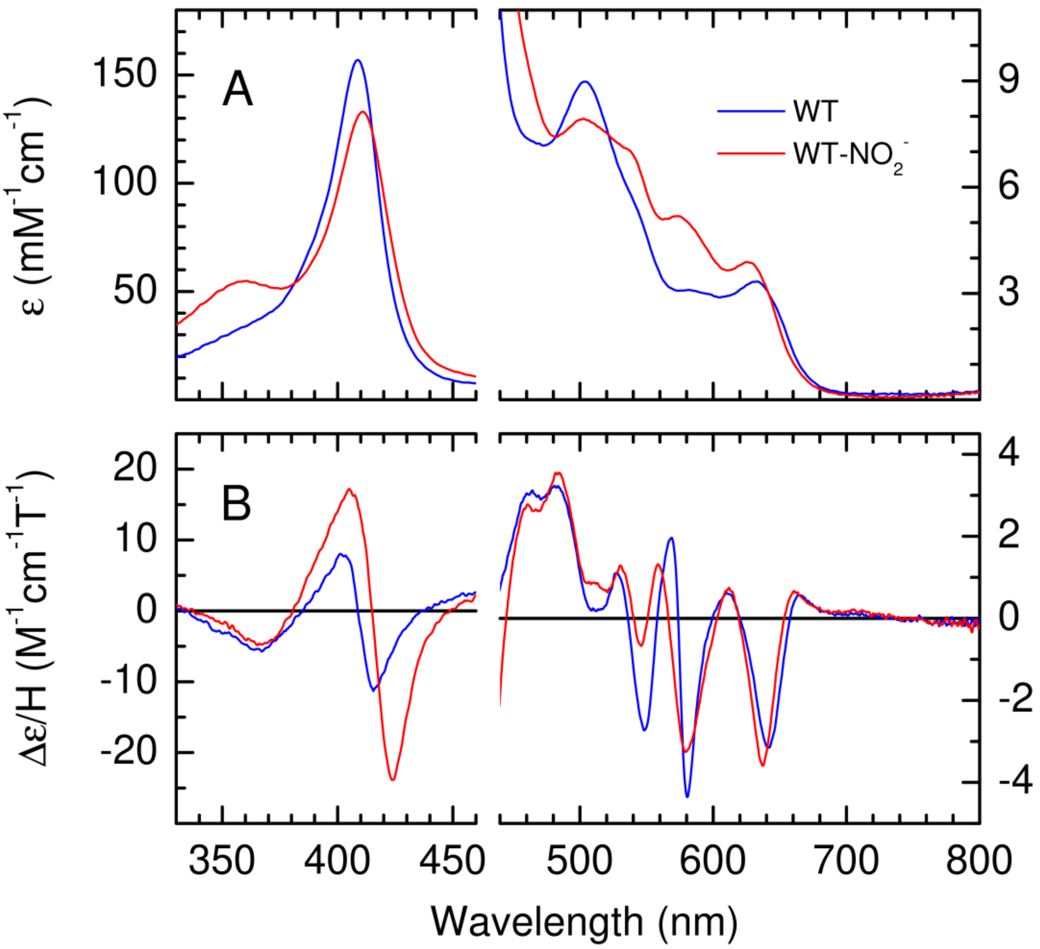

Figure S1. Spectroscopic changes induced by the addition of 100 mM nitrite to wild-type Sperm Whale myoglobin. **Panel A:** UV/vis electronic absorption spectrum **Panel B:** UV/vis RT-MCD spectrum before (790 μM blue trace) and after (640 μM red trace)

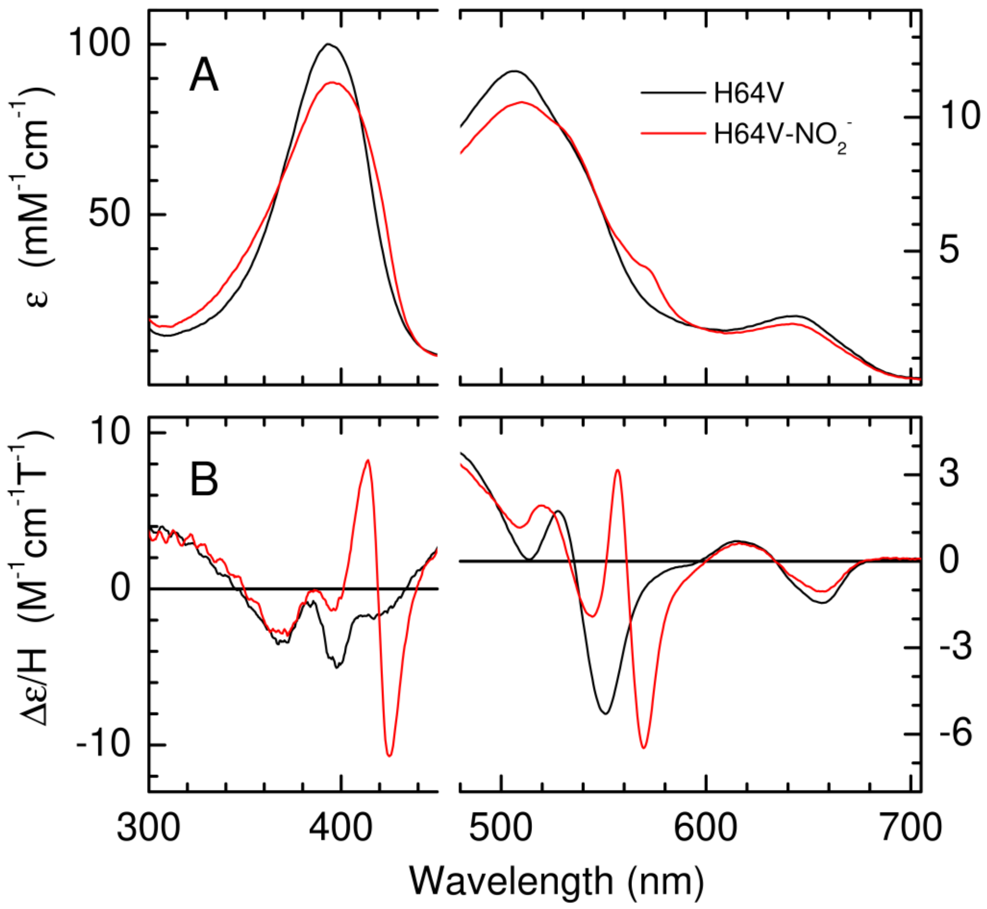

Figure S2 Spectroscopic changes induced by the substitution of the distal histidine ligand (His-64) by valine in Fe (III) Sperm Whale myoglobin in the absence and presence of nitrite **Panel A**: UV/vis electronic absorption spectrum **Panel B**: UV/vis RT MCD spectrum

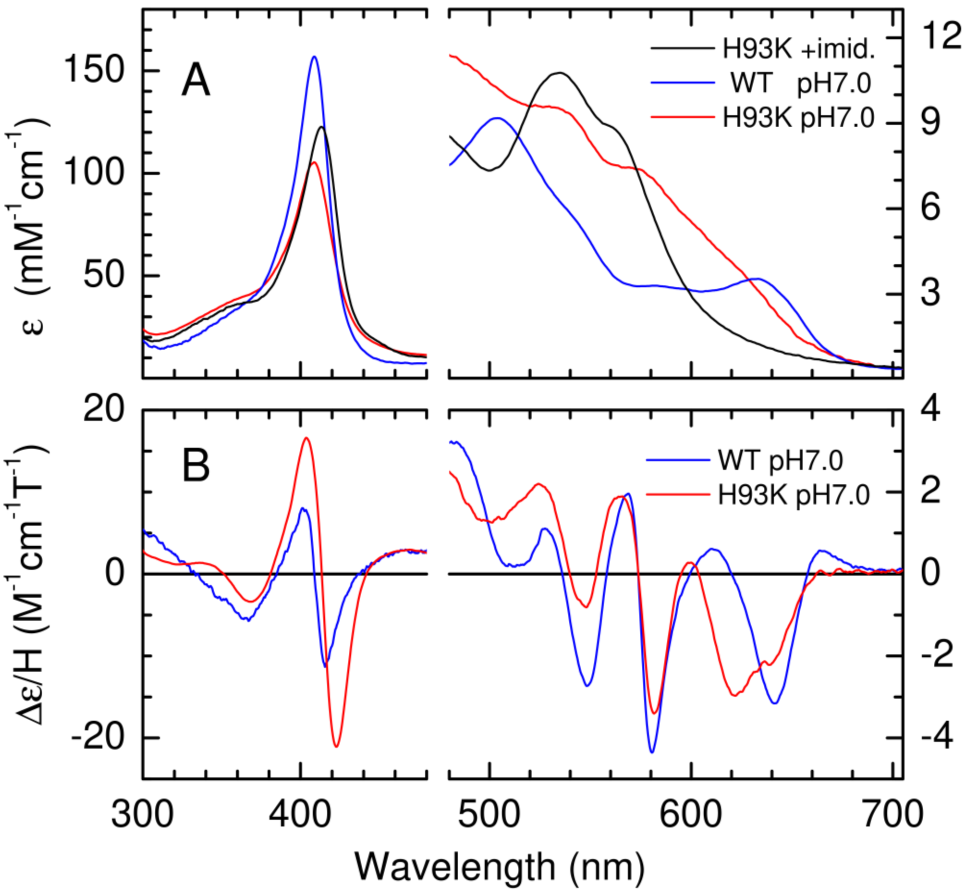

Figure S3. Spectroscopic changes induced by the substitution of the proximal histidine ligand (His-93) by lysine in Fe(III) Sperm Whale myoglobin in the absence and presence of imidazole. **Panel A:** UV/vis electronic absorption spectrum **Panel B:** UV/vis RT-MCD spectrum

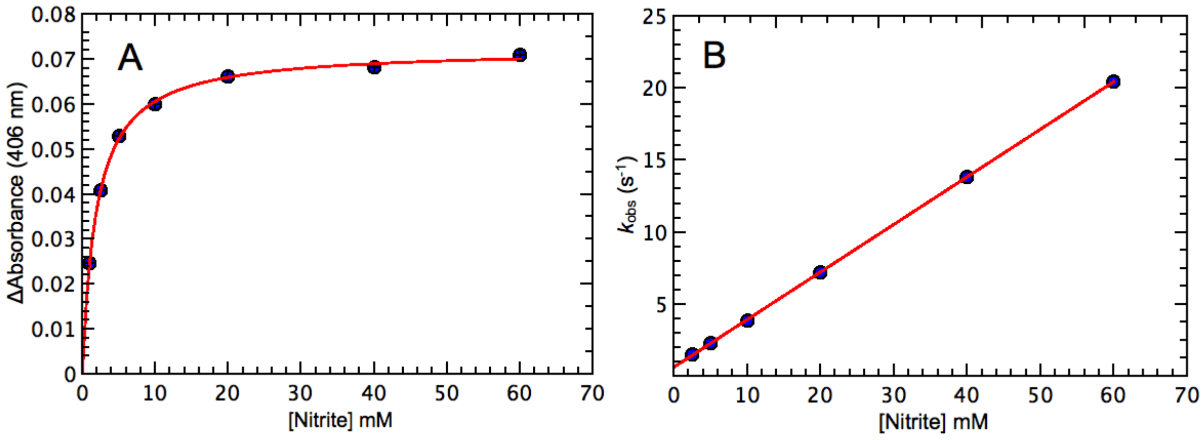

Figure S4. Concentration dependence of the reaction of the Fe (III) form of the H93K variant of Sperm Whale myoglobin with nitrite

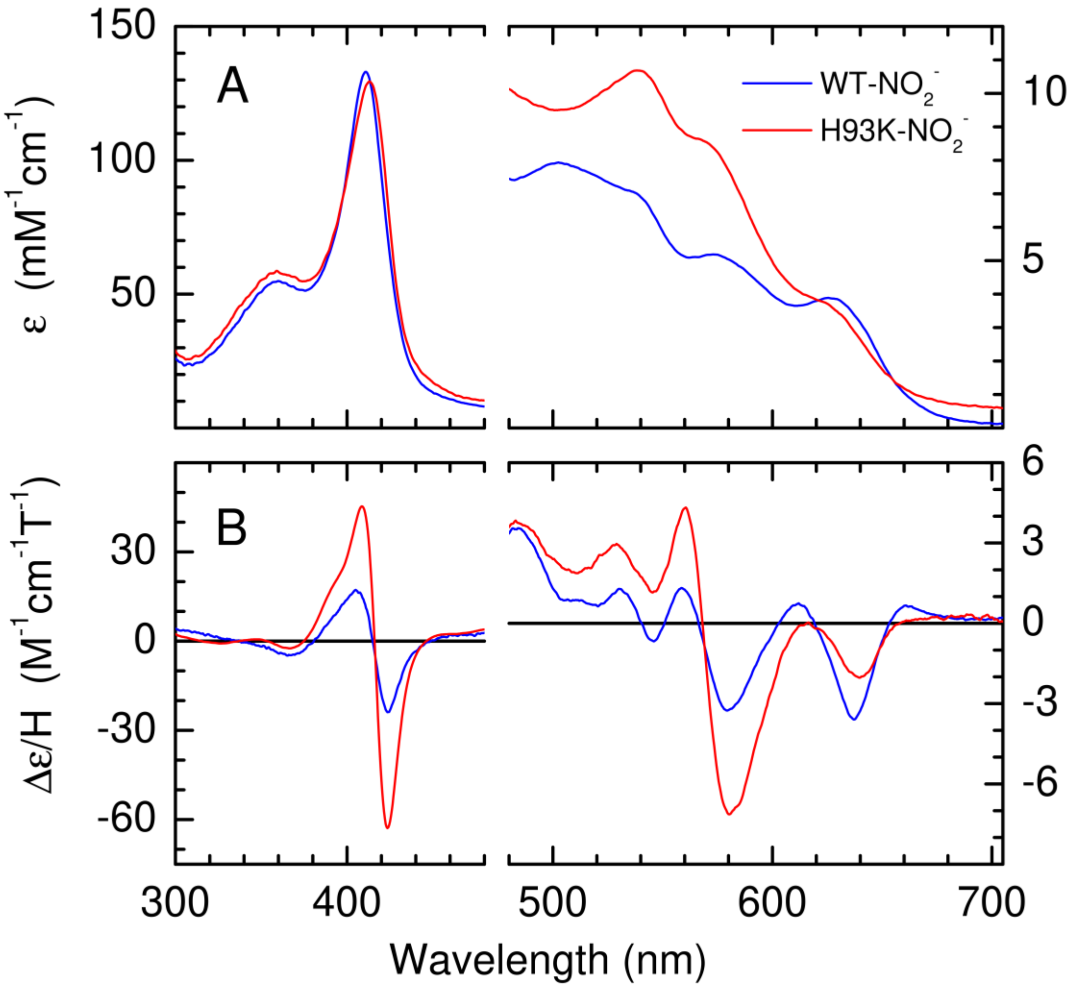

Figure S5 Spectroscopic changes induced by the substitution of the proximal histidine ligand (His-93) by lysine in Fe (III) Sperm Whale myoglobin in the presence of nitrite Panel A UV/vis electronic absorption spectrum Panel B UV/vis RT MCD spectrum

Oxidation/ligand State	Wild-type Sperm Whale Myoglobin		H93K Sperm Whale Myoglobin	
	Soret Maximum	Visible region	Soret Maximum	Visible region
Fe (III)	409 nm	Max 503, 596 & 642 nm	408 nm	Max 498, 542, 575 & *ca* 625 nm
Fe (II)-NO^+	420 nm	Max. 574,535 nm<br>Min	420 nm	Max. 570,534 nm<br>Min
Fe (II)	434 nm	Max. 557 nm	429 nm	565 nm
Fe (II)-NO	421 nm	Max. 581,549 nm<br>Min.	419 nm	Max. 576,545 nm<br>Min

Table S1 Summary of the key features of the UV/vis electronic absorption spectra of wild-type and H93K Sperm Whale myoglobin in the absence and presence of bound NO.
